# Quality of life and associated factors on employees of a public university working remotely during the COVID-19 pandemic

**DOI:** 10.47626/1679-4435-2022-808

**Published:** 2022-03-30

**Authors:** Igor Garcia Barreto, Ramayana e Silva Costa, Patrick Mac’Donald Farias Pires de-Oliveira, Aline Santiago Barbosa, Taise de Oliveira da-Silva

**Affiliations:** 1 Núcleo de Gestão e Atenção à Saúde e Segurança do Trabalho, Universidade Federal do Recôncavo da Bahia, Cruz das Almas, BA, Brazil.

**Keywords:** quality of life, risk factors, occupational health, COVID-19, remote work

## Abstract

**Introduction:**

The pandemic caused by the severe acute respiratory syndrome coronavirus 2 imposed restrictions to movement, generating new work dynamics especially in the education sector, where remote working has become the rule. The overlapping of work-related and domestic tasks and the fear of the virus generated an additional burden to workers, with unknown effects on their quality of life.

**Objectives:**

To estimate the quality of life of employees of the education sector who were working remotely during the pandemic caused by the severe acute respiratory syndrome coronavirus 2 and identify associated factors.

**Methods:**

This is a cross-sectional study performed with a sample of 317 government employees of a federal university between August 25 2020 and September 11 2020. Standardized questionnaires concerning sociodemographic and economic aspects were constructed using the Google Forms tool. The European Health Interview Survey - Quality of Life 8-item index was used to assess quality of life. Multiple linear regression was used to check for associations between variables using quality of life scores as outcome (alpha value of 5%). This research proposal was approved by the National Research Ethics Commission, with a Certificate of Presentation for Ethical Appreciation No. 33636220.1.0000.0056.

**Results:**

The European Health Interview Survey - Quality of Life 8-item index resulted in mean adjusted scores of 3.5 ± 1.9. Quality of life was independently associated with age (β = 0.01, 95% confidence interval 0.00 to 0.02, p = 0.015), physical activity (β = 0.19, 95% confidence interval 0.00 to 0.38, p = 0.049), smoking habits (β = 0.54, 95% confidence interval 0.19 to 0.88, p = 0.002), having a dedicated workspace (β = 0.14, 95% confidence interval 0.02 to 0.26, p = 0.023), performing housework (β =-0.20, 95% confidence interval-0.32 to-0.08, p < 0.001), financial difficulties (β =-0.26, 95% confidence interval-0.40 to-0.12, p < 0.001), and the impact of social distancing at work (β =-0.33, 95% confidence interval-0.47 to-0.19, p < 0.001).

**Conclusions:**

The level of quality of life within the sample was reasonable; it was higher among older participants who were physically active and did not smoke, and lower when the socioeconomic situation was unfavorable. This highlights the importance of constructing support strategies while the effects of the pandemic linger.

## Introduction

At the end of 2019, a new coronavirus was identified and named severe acute respiratory syndrome coronavirus 2 (SARS-CoV-2), being responsible for cases of pneumonia in the city of Wuhan, Hubei province, China. It soon spread worldwide, triggering a pandemic recognized by the World Health Organization (WHO) in March 11 2020. Its high contagiousness, significant probability of causing severe disease in humans (COVID-19), and the lack of vaccines or effective treatment strategies at the time^1^ led public authorities of many countries to adopt restrictive measures, such as social distancing or even lockdowns.^2^

In Brazil, the first case was reported in February 26, 2020. In spite of the absence of a national plan for fighting COVID-19 and minimizing its impacts by some of the country’s representatives, a large part of the country adopted control measures such as the closure of nonessential businesses and promotion of social distancing and remote work, in addition to the mandatory use of masks in public areas and encouragement of handwashing and hand sanitizer use.^3^ The virus soon spread throughout the country, reaching 19,089,940 cases of the disease and 533,488 deaths by July 2021.^4^

Social distancing, which can be defined as “keeping a safe space between yourself and other people who do not live with you,”^5^ generated new dynamics of personal and professional relationships where remote work gained relevance.^6^ This was especially important in the education sector due to characteristics inherent to its activities, which tend to create gatherings and consequently spread the virus. This led to the generalized and sudden closure of educational institutions, thus affecting the routine of workers of this sector. At federal level, for example, 95% of government employees related to education switched to remote work, whereas in other government bodies this happened with only 49% of the workers.^6^

According to the WHO, quality of life (QoL) can be defined as “individuals’ perceptions of their position in life in the context of the culture and value systems in which they live and in relation to their goals, expectations, standards, and concerns.”.^7^ This is a quite broad concept, being affected by various aspects of an individual’s life, such as physical health, psychological state, personal beliefs, and social relations.^7^ With social distancing and the progression of the pandemic, together with the possibility of increases in domestic and remote workloads — the latter being associated with a salary reduction for 58.9% of people^8^ —, the fear of getting sick, the need for greater interaction among family members, and fear of unemployment, one should expect a considerable increase in the mean family burden.^9^ Despite these data, the effect of this tension on the QoL of individuals working remotely is still uncertain.

Most studies on this subject assessed the impact of QoL in the general population, in specific groups of workers such as healthcare professionals, or in groups of people with a specific morbid condition. Tran et al.^10^ assessed QoL in the general population in Vietnam during social distancing and found lower levels of QoL among people with chronic diseases and limited-term employment contracts; they also found lower levels of self-perceived health among women, people with chronic diseases and who lived in families of three to five people. Suryavanshi et al.^11^ assessed mental health and QoL among healthcare professionals during the pandemic in India. They found a low QoL in 45% of the interviewees; depression, moderate anxiety, or severe anxiety were independently associated with low QoL. Ping et al.^12^ assessed QoL in the general population in China during the pandemic and found lower QoL scores among retired people, older people, those with chronic diseases, and those who were worried about catching COVID-19. Despite data available in the literature on this subject, we have not yet identified information on the effect of social distancing on the QoL of workers of the education sector, where remote work became the rule.

This study aimed to assess health-related QoL among employees of a higher education institution who were working remotely. Secondly, factors associated with a better QoL in the study population were assessed with the aim of improving support strategies while the effects of the pandemic linger.

## Methods

### Sample

We conducted a cross-sectional cohort study at Universidade Federal do Recôncavo da Bahia (UFRB). This institution, with a multi-campus structure, is located in the inland region of the state of Bahia and has 1,589 active employees; these encompass 892 professors and 697 administrative assistants (AA). Recruitment took place from August 25 2020 and September 11 2020. During this period, all in-person activities were suspended as a restrictive measure due to the public health issue caused by the pandemic, and all its employees were working remotely.

The workers were invited to participate in the study through the institution’s official webpage, professional email addresses, and social media. Individuals who accepted to participate in the study were directed to a structured questionnaire to be answered online via the Google Forms platform. This study was approved by the National Research Ethics Commission (CONEP) under Certificate of Presentation for Ethical Appreciation (CAAE) No. 33636220.1.0000.0056; all participants agreed with the online Informed Consent Form (ICF). The study was then performed with a convenience sample consisting of 317 government employees, of which 131 were professors and 186 were AA who accepted to participate in the study and answered the questionnaire.

### Measures

After accessing the platform, reading the online ICF, and accepting to participate in the study, individuals were automatically redirected to the study questionnaires. The questionnaire was divided into four parts, involving the following themes: demographic, social, and economic aspects, and QoL. Information such as age, gender, position, physical activity (PA), smoking habits, and alcohol intake were collected, in addition to socioeconomic aspects such as income and matters related to family and living conditions. The “marital status” variable was categorized as “single,” “married or cohabiting,” “divorced or separated,” and “widowed.” Race/skin color was classified according to criteria by the Brazilian Institute of Geography and Statistics (IBGE) as “White,” “Black,” “Brown,” “Yellow,” and “Indigenous.” Schooling was classified into five categories (“secondary education or lower,” “undergraduate education,” “graduate education,” “masters,” and “doctorate or higher”). Alcohol intake was divided into “does not consume,” “occasionally consumes,” “consumes on the weekends,” and “consumes daily.” Smoking habits classified individuals into “non-smokers,” “former smokers,” and “current smokers.” Guidelines by the American College of Sports Medicine (ACSM) were used for defining physically active individuals as those who had 30 minutes or more of moderate PA at least 5 days a week or 20 minutes of vigorous PA at least 3 days a week; the remaining participants were classified as having a sedentary lifestyle.^13^

The European Health Interview Survey - Quality of Life 8-item index (EUROHIS-QoL-8) instrument was used for measuring QoL. It contains eight questions that assess physical, psychological, social, and environmental domains. This instrument is a short form of the World Health Organization Quality of Life Instrument - Abbreviated version (WHOQOL-Bref), containing two general questions and six questions encompassing the domains mentioned above. The answers follow a Likert scale and their mean value represents a global QoL score ranging from 1 to 5, where higher values correspond to a higher QoL. The instrument was also translated and validated in Brazilian Portuguese.^14^

### Statistical analysis

Variables were descriptively presented as absolute numbers and proportions. Descriptive data and a univariate analysis were stored and analyzed using SPSS version 23.0 for Windows (SPSS Inc., Chicago, EUA). A multivariate analysis was performed using R, version 3.6.3 (R Foundation for Statistical Computing, Vienna, Austria). First, a t-test and a chi-squared test were applied for assessing sample representativeness within the university population, where correction factors were used for adjusting EUROHIS scores. Categorical variables were described as absolute values and percentages regarding the sample, whereas numerical values were described as means and standard deviations or median values and interquartile ranges, according to each case.

After the descriptive stage, further analyses aimed to verify possible factors associated with QoL scores among employees who were working remotely. For this, QoL was used as a dependent variable, while sociodemographic and economic data were independent variables. First, univariate analyses were performed using a simple linear regression. Then, a multivariate analysis using multiple linear regression was performed, where a backward elimination process successively excluded less significant variables until reaching a p-value < 0.1, which was used as threshold for the inclusion of variables in the final model. Both in the univariate model and in the final multivariate model, variables were considered statistically significant if p < 0.05.

## Results:

Information on the assessment of sample representativeness are shown in Table S1 (online-only supplementary material). Since gender, career, and schooling presented significant differences, they were used for correcting the QoL scores, resulting in a mean adjusted score of 3.5 ± 1.9.

Column 2 of Table 1 presents the characteristics of the sample. The mean age of participants was 41.6 ± 7.9 years; most of them were female (60.9%), married (58.9%), Brown (44.6%), non-disabled (94%), held an AA position (58.7%), and had a doctorate degree (36.7%). Regarding lifestyle habits, 88.3% had a sedentary lifestyle according to the ACSM criteria, 93.4% were non-smokers, and 48.6% occasionally consumed alcoholic beverages.

**Table 1:** Characteristics of the sample and univariate analysis of factors associated with QoL among government employees of Universidade Federal do Recôncavo da Bahia (n = 317)

Variables	n (%)	QoL ( $\bar{x}\pm SD$ )	Unstandardized β (95%CI)*	p-value
Age ( $\bar{x}\pm SD$ )	41.6 ± 7.9		0.01 (0.01/0.02)^†^	0.015
< 41 years		3.36 ± 0.56		
≥ 41 years		3.50 ± 0.60		
Gender^‡^				0.078
Female	193 (60.9)	3.39 ± 0.56	Ref.	
Male	123 (38.8)	3.51 ± 0.61	0.12 (-0.01/0.25)	
Marital status				0.114
Single	101 (32.0)	3.42 ± 0.59	Ref.	
Married or cohabiting	186 (58.9)	3.48 ± 0.57	0.06 (-0.08/0.20)	
Divorced or separated	25 (7.9)	3.20 ± 0.59	-0.22 (-0.47/0.03)	
Widowed	4 (1.3)	3.22 ± 0.53	-0.20 (-0.78/0.38)	
Skin color				0.058
White	99 (31.5)	3.53 ± 0.47	Ref.	
Brown	140 (44.6)	3.40 ± 0.61	-0.13 (-0.28/0.02)	
Black	69 (22.0)	3.36 ± 0.58	-0.14 (-0.35/0.00)	
Yellow	4 (1.3)	2.84 ± 0.41	-0.69 (-1.25/-0.12)	
Indigenous	2 (0.6)	3.62 ± 0.88	0.09 (-0.70/0.88)	
Position				0.928
Professor	131 (41.3)	3.44 ± 0.57	Ref.	
Administrative assistant	186 (58.7)	3.43 ± 0.58	-0.01 (-0.14/0.12)	
Physical activity^§^				0.012
Active	37 (11.7)	3.65 ± 0.50	0.25 (0.06/0.44)	
Sedentary	278 (88.3)	3.40 ± 0.58	Ref.	
Smoking habits				0.011
No	296 (93.4)	3.46 ± 0.57	0.49 (0.15/0.84)	
Yes	11 (3.5)	3.24 ± 0.38	0.27 (-0.22/0.76)	
Former smoker	10 (3.2)	2.96 ± 0.59	Ref.	
Alcohol intake				0.404
Does not consume	91 (28.7)	3.44 ± 0.52	0.48 (-0.10/1.06)	
Occasionally	154 (48.6)	3.43 ± 0.58	0.46 (-0.12/1.03)	
At the weekends	67 (21.1)	3.47 ± 0.63	0.50 (-0.08/1.09)	
Daily	5 (1.6)	2.97 ± 0.62	Ref.	
Schooling				0.885
Secondary education or lower	15 (4.7)	3.46 ± 0.97	Ref.	
Undergraduate education	22 (7.0)	3.39 ± 0.51	-0.07 (-0.45/0.32)	
Graduate education	101 (32.0)	3.40 ± 0.51	-0.06 (-0.37/0.26)	
Masters	62 (19.6)	3.49±0.61	0.03 (-0.29/0.36)	
Doctorate or higher	116 (36.7)	3.44 ± 0.57	-0.01 (-0.33/0.30)	
Has a disability?				0.835
Yes	19 (6.0)	3.41 ± 0.59	-0.03 (-0.30/0.24)	
No	296 (94)	3.44 ± 0.58	Ref.	
Is the main caretaker of children?				0.119
Yes	147 (48.2)	3.38 ± 0.58	-0.10 (-0.23/0.03)	
No	158 (51.8)	3.49 ± 0.57	Ref.	
		Continued on next page		
Is the main family member to perform housework?				< 0.001
Yes	184 (59.2)	3.34 ± 0.56	-0.24 (-0.37/-0.17)	
No	127 (40.8)	3.59 ± 0.57	Ref.	
Per capita income (R$)				0.227
< 2,000.00	65 (22.1)	3.35 ± 0.67	-0.15 (-0.34/0.04)	
2,000.00 to 3,249.99	81 (27.6)	3.37 ± 0.51	-0.14 (-0.32/0.04)	
3,250.00 to 4,999.99	74 (25.2)	3.45 ± 0.60	-0.01 (-0.19/0.18)	
≥ 5,000.00	74 (25.2)	3.51 ± 0.53	Ref.	
Is having financial difficulties during the pandemic?				< 0.001
Yes	76 (24.1)	3.21 ± 0.57	-0.30 (-0.45/-0.16)	
No	240 (75.9)	3.51 ± 0.56	Ref.	
Has a dedicated workspace in the household?				< 0.001
Yes	157 (49.7)	3.53 ± 0.58	0.17 (0.05/0.30)	
No	159 (50.3)	3.35 ± 0.56	Ref.	
Owns a computer?				0.043
No	21 (6.6)	3.22 ± 0.67	-0.13 (-0.41/0.14)	
Yes, only user	217 (68.5)	3.49 ± 0.56	0.13 (-0.01/0.28)	
Yes, shares with family members	79 (24.9)	3.35 ± 0.58	Ref.	
How did remote work impact your workload?				0.467
Decreased workload	60 (19.0)	3.45 ± 0.61	0.05 (-0.12/0.22)	
Kept the same	102 (32.3)	3.49 ± 0.58	0.09 (-0.05/0.24)	
Increased workload	154 (48.7)	3.40 ± 0.56	Ref.	
How often is your remote work interrupted due to other reasons?				< 0.001
Never	46 (14.6)	3.66 ± 0.64	Ref.	
1-3 times/day	150 (47.8)	3.50 ± 0.54	-0.16 (-0.34/0.03)	
> 3 times/day	67 (21.3)	3.35 ± 0.53	-0.31 (-0.52/-0.10)	
Always	51 (16.2)	3.19 ± 0.57	-0.47 (-0.70/-0.25)	
How is social isolation affecting your work activities?				< 0.001
Made it more difficult	162 (52.3)	3.28 ± 0.56	-0.36 (-0.50/-0.21)	
Kept the same	61 (19.7)	3.62 ± 0.55	-0.02 (-0.20/0.16)	
Contributed to it	87 (28.1)	3.64 ± 0.53	Ref.	

SD = standard deviation; 95%CI = 95% confidence interval; QoL = quality of life; Ref. = reference category.

* Unstandardized β coefficients represent the variation in mean QoL scores between the reference category and the current category.

† These values represent the mean variation in QoL scores for each 1-year increment in age.

‡ The “non-binary” category was excluded due to the lack of representation.

§ According to criteria by the American College of Sports Medicine (ACSM).

Regarding socioeconomic characteristics (Table 1), 48.2% and 59.2% of the participants were the main caretakers of a family member or the main responsible for housework, respectively. It is important to highlight a gender disparity regarding this last variable, since 76.8% of women stated being the main responsible for housework, whereas only 30.8% of men mentioned this answer. The mean per capita income was R$ 3,250.00 (interquartile range 2,000.00-5,000.00). Although the participants’ income did not significantly decrease, 24.1% of the individuals stated that they were having financial difficulties due to the pandemic.

As to remote work, most participants (68.5%) said they had their own computer and did not share it with their family members, and 50.3% did not have a dedicated workspace in their household. Altogether, 48.7% of all participants considered that their workload increased with the pandemic: 75.6% of the professors and 29.7% of the AA. Most individuals (47.8%) stated that they had to interrupt work one to three times a day due to responsibilities not related to work, and 52.3% considered that social isolation made the execution of their work activities more difficult.

In the simple linear regression model (Table 1), associations were observed for age (β = 0.01, p = 0.015), PA (β = 0.25, p = 0.012), smoking habits (β =-0.49, p = 0.011), housework (β =-0.24, p < 0.001), financial difficulties (β =-0.30, p < 0.001), having a dedicated workspace (β = 0.17, p < 0.001), owning a computer (β = 0.13, p = 0.043), frequency of interruptions due to other reasons (β =-0.47, p < 0.001), and finally, the impact of social isolation on work demands (β =-0.36, p < 0.001).

Table 2 shows the multivariate linear regression model. Age (β = 0.01, p = 0.015), PA (β = 0.19, p = 0.049), smoking habits (β = 0.54, p = 0.002), housework (β =-0.20, p < 0.001), financial difficulties (β =-0.26, p < 0.001), having a dedicated workspace (β = 0.14, p = 0.023), and the impact of social isolation on work demands (β =-0.33, p < 0.001) were independently associated with QoL.

**Table 2:** Multivariate analysis of factors associated with QoL among government employees of Universidade Federal do Recôncavo da Bahia (n = 317)

Variables	Standardized β (95%CI)*	p-value
Age ( $\bar{x}\pm SD$ )	0.01 (0.00/0.02)^†^	0.015
Physical activity^‡^		
Active	0.19 (0.00/0.38)	0.049
Sedentary	Ref.	
Smoking habits		
No	0.54 (0.19/0.88)	0.002
Yes	0.31 (-0.18/0.81)	0.217
Former smoker	Ref.	
Has a dedicated workspace in the household?		
Yes	0.14 (0.02/0.26)	0.023
No	Ref.	
Is the main family member to perform housework?		
Yes	-0.20 (-0.32/-0.08)	< 0.001
No	Ref.	
Is having financial difficulties during the pandemic?		
Yes	-0.26 (-0.40/-0.12)	< 0.001
No	Ref.	
How is social isolation affecting your work activities?		
Made it more difficult	-0.33 (-0.47/-0.19)	< 0.001
Kept the same	-0.05 (-0.23/0.12)	0.538
Contributed to it	Ref.	

SD = standard deviation; 95%CI = 95% confidence interval; QoL = quality of life; Ref. = reference category.

* Unstandardized β coefficients represent the variation in mean QoL scores from the reference category to the current category.

† These values represent the mean variation in QoL scores for each 1-year increment.

‡ According to criteria by the American College of Sports Medicine (ACSM).

## Discussion

This study offers a perspective on the QoL of workers of the education sector who were working remotely due to social distancing measures enforced during the COVID-19 pandemic. Firstly, we identified reasonable QoL scores among the participants (*x* = 3.5 ± 1.9). In this context, sociodemographic and economic factors, as well as lifestyle habits, were independently associated with QoL. Secondly, we observed a higher QoL among individuals who were older, physically active and non-smokers. Thirdly, stressors connected to the socioeconomic and environmental domains also played an important role, interfering negatively with the individual perception of QoL.

QoL can be understood as an individual perception of people. However, it is important to note that despite these perceptions, QoL depends on and involves many factors, which are individual and collective, subjective and objective/material. In this study, we found QoL scores of 3.5 in the sample of workers, demonstrating a reasonable QoL during social distancing. These findings are in line with other studies performed with education professionals, where QoL scores standardized with the scale used in this study varied from 3.2 to 3.8.^15,16^ It should be noted that these studies only evaluated professors, excluding other professionals involved with the teaching and learning process; in addition, they were performed outside of the pandemic context. Studies performed in the general population subjected to restrictions due to COVID-19 also showed varied results: Azizi et al.^17^ and Wang et al.^18^ found standardized QoL scores of 4.3 and 3.14, respectively.

Age was revealed to be an important factor associated with QoL, both in unadjusted and adjusted analyses, showing that QoL increases progressively with age. Different results have been found in most studies, considering international literature and studies conducted in the general population during the pandemic. Azizi et al.^17^ and Kharshiing et al.^19^ did not find statistically significant differences between age and QoL, whereas Ping et al.^12^ observed that QoL was inversely proportional to age. These differences can be explained by the fact that these studies were performed in the general population and with lower mean ages than in this study (33.19, 31.8, and 38.3 years, respectively). The study by Ping et al. reported a higher variation in age (12 to 78 years) in comparison with our study (26 to 70 years, mean = 41.6), which was performed with active employees, and, in this case, age can also be associated with other factors that justify a higher self-perception of QoL such as salary, housing, and job stability. Considering the specificities of the study population, Sanchez et al.^15^ also assessed QoL among professors and found a positive relationship between age and QoL.

Although a significant amount of workers mentioned they performed a certain degree of PA, we found a very low proportion of individuals considered physically active during the validity of social distancing measures. This number was much lower than in a previous study performed in the same population using the same criteria for defining a person as physically active^20^ — we found a proportion of 11.7% of physically active persons, whereas 47.7% of individuals in the study were considered active. This discrepancy might have had a negative impact on people’s QoL, since individuals who remained active had a mean QoL score that was 0.19 higher than the others. It is expected that people in home isolation (as a way to contain the spread of SARS-CoV-2) change their lifestyle habits, including a reduction in their usual PA habits.^21^ Conversely, this change can have negative consequences for some diseases such as hypertension, diabetes, and respiratory diseases,^21^ which are also risk factors for severe COVID-19.^1^ Our findings were in accordance with other studies on this theme that also observed a decrease in the level of PA during the pandemic^17,18,22^ and better QoL among those who remained physically active.^22^

Smoking habits were associated with QoL in the studied sample: non-smokers had a mean QoL score that was 0.54 points higher than that of former smokers. When comparing this last category with current smokers, no relationship was observed. On the other hand, the literature reports worse QoL among smokers and a significant improvement of QoL in those who quit this habit.^23^ This could be explained as in this study we did not evaluate under which circumstances smoking cessation happened among self-declared former smokers, such as the previous level of exposure to tobacco, reason for cessation, and time since quitting. This lack of data hinders a deeper understanding of this subject, since former smokers may have been intense smokers who quit only recently due to the pandemic and fear of contracting severe forms of the disease. As it is common knowledge, smoking is a risk factor for severe COVID-19, increasing mortality by this disease.^24^

Considering the existence of a dedicated workspace at home, we also identified an association with QoL, since participants who declared having such a space had a mean QoL that was 0.14 points above those who did not have it. This implies, among other matters, that the transition to remote work in this university due to the pandemic happened abruptly, in many cases causing workers to invest in supplies for facilitating their work activities, since no specific resources were allocated to structuring this work modality. This was well documented by Souza,^8^ who presented data on telework among Brazilian workers (a work modality that is similar to remote work, except for the fact that it is regulated): despite the potential of this work modality, considering that 65% of scientists and intellectuals and 61% or administrators/managers in Brazil are capable of performing telework,^25^ its abrupt implementation generated overlapping with other activities. Corroborating this statement, 50.3% of the workers in our study declared not having a dedicated workplace at home.

As to other activities overlapping with remote work, we identified that individuals who declared being the main family member to perform housework had a mean QoL that was 0.20 points lower than the others. The need to remain productive during remote work overpowers the responsibility of doing housework, which on top of that are unpaid. This probably contributes to a greater difficulty in finding time for self-care and leisure activities. Furthermore, although this is not the focus of our work, it cannot be ignored that women are predominantly the ones who perform housework considering the sexual division of labor.^26^ Future studies performed in other populations or with larger samples could further clarify this aspect.

In addition to these variables, we also identified that those who had financial difficulties due to the pandemic had lower mean QoL scores (0.26 points lower than those who did not). It is known that the financial situation is directly related with the material maintenance of life, whether it be for acquiring supplies for subsistence or providing leisure. Although government employees have job stability and higher mean salaries than those in the private sector,^27^ this does not mean that these resources are sufficient for their maintenance and, at times, that of their families. Situations involving government employees who are in debt due to the ease of access to purchases and loans and to the fact that many of them are the main providers for their families are widely known. In addition to these factors, it is important to highlight that, due to the remote work situation, some benefits were suspended.^28^ Furthermore, from the moment the employees started performing their activities at home, they were the ones responsible for the costs of remote work (such as electricity and equipment), since the employer did not support this new work modality.^29^

Finally, individuals who stated that social distancing made the execution of their work activities more difficult presented a QoL score that was 0.33 points lower when compared to those who said that social distancing was positive. It is worth noting that social isolation, by hampering in-person contact with colleagues, and the change in environment itself can to a certain extent generate a production process that is solitary and more individualized. This could also compromise the collective articulation that implicates, among other aspects, in the revindication of rights and improved working conditions. New work settings imposed to workers by the pandemic can generate instability between the need to adapt to a new way of working and one’s personal routine. All this, at times added to a work overload and difficulties in managing personal time, can lead to psychological illness.^30^

We highlight that this study explored QoL and its associated factors in a relatively large sample (N = 317) of active workers of the education sector who were affected by the COVID-19 pandemic, switching to remote work. Due to the social distancing measures enforced at the time, although this study was performed at an educational institution, the recruitment stage was challenging and performed exclusively via electronic means; problems with access thus did not allow us to reach a higher number of participants. This was reflected in the response rate to the invitation: answers were obtained from approximately 20% of the institution’s 1,589 workers, or 14.7% of professors and 26.7% of AA. In spite of the techniques used for correcting QoL scores, some degree of selection bias cannot be excluded from the present study. Since we used a cross-sectional design, we cannot make inferences regarding causality. There were also no data referring to most of these variables in studies prior to the social isolation period for a better understanding of the effects of the pandemic in the studied population. Future longitudinal studies that follow this population until a post-pandemic time could increase the accuracy of this information.

## Conclusions

In this study, we assessed QoL and its associated factors in a sample of workers of a higher education institution who were working remotely during the COVID-19 pandemic. We found reasonable levels of QoL among the participants, despite the restrictive measures enforced in this period. In a multivariate model, we observed that older individuals who remained physically active, non-smokers, and those with a dedicated workspace at home had higher mean QoL scores. On the other hand, factors such as simultaneous housework, financial difficulties, and difficulties at work caused by social distancing had a negative impact on QoL. Considering the data presented here, work conditions and relations cannot be dissociated from the employees’ QoL; strategies aimed at health care and attention, especially those that can be performed remotely, are thus fundamental. For this, the creation of safety protocols, informative and preventive actions, and actions aimed at health promotion deserve greater attention.

Finally, we highlight that the COVID-19 pandemic happened on top of the loss of resources being experienced by educational institutions in recent years, highlighting the need to further reflect on work settings, especially considering the deepening of its precarization, in addition to the consequences to workers’ QoL.
